# Diagnosis of Undifferentiated Connective Tissue Disease in a Patient With Digital Gangrene and Positive Antinuclear Antibodies

**DOI:** 10.7759/cureus.15883

**Published:** 2021-06-24

**Authors:** Pradeep Ravi, Molly Mary Thabah, Rohan J Verghese, Sekar Dineshbabu, Tamilarasu Kadhiravan

**Affiliations:** 1 Medicine, Jawaharlal Institute of Postgraduate Medical Education and Research, Puducherry, IND

**Keywords:** gangrene, ischemia, lupus, systemic lupus erythematosus, uctd, connective tissue disorder

## Abstract

The occurrence of ischemia of the digits or digital gangrene is a well-known complication of systemic autoimmune diseases, such as systemic sclerosis, systemic lupus erythematosus, and anti-phospholipid syndrome, among others. The pathophysiological mechanisms are small vessel vasculitis, vasospasm of Raynaud’s phenomenon, microthrombi due to antiphospholipid syndrome, and/or accompanying accelerated atherosclerosis. Digital ischemia can also occur in the context of disseminated bacterial infections and sepsis. We present here the case of a patient who had digital ischemia and positive antinuclear antibodies but without well-defined clinical features of a connective tissue disease. A diagnosis of undifferentiated connective tissue disease was made.

## Introduction

Occurrence of gangrene of the fingers and toes is a symptom of vascular insufficiency arising due to various causes, such as atherosclerosis, infections, coagulation abnormalities [1], vasculitis [2], or an autoimmune disease [3]. Identification of the cause of gangrene is important as the treatment can change and the consequence of gangrene can be devastating to the patient. Among the connective tissue diseases, systemic sclerosis, systemic lupus erythematosus, and antiphospholipid syndrome are known to cause digital gangrene [3,4]. We present here the case of a patient who presented with gangrene of the fingers, was positive for antinuclear antibodies (ANA) but did not have any other features of a connective tissue disease.

## Case presentation

**Figure 1:**
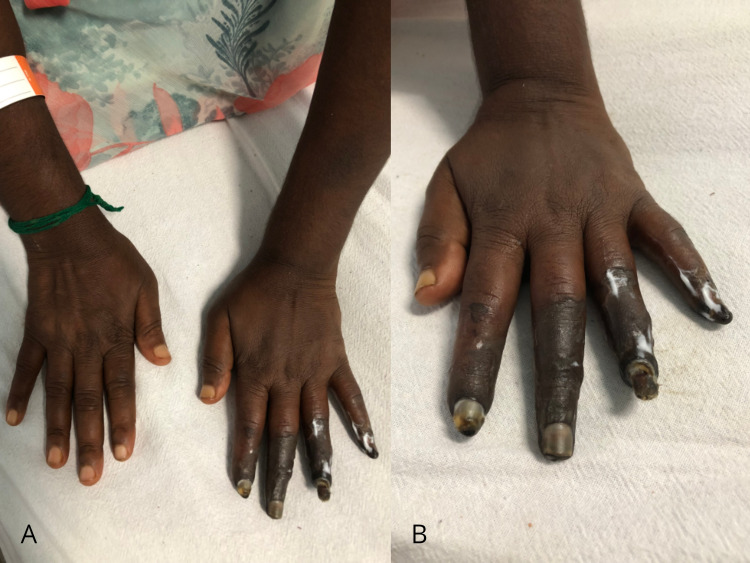
A picture showing gangrene of the fingers of the left hand except for the thumb. Panel A shows both hands. Note that the right hand is normal. Panel B is the left hand with gangrene of the fingers.

A 45-year-old woman presented with pain and ulceration over the index and ring finger of the left hand for one month, followed by blackening of index, ring, and little finger of left hand over one week. There were similar lesions over the right second and fourth toe. There was no history of diabetes, hypertension, Raynaud’s phenomenon, arthralgia, dysphagia, oral ulcers, or fever. On examination, she was conscious and alert. Pulse was regular, all peripheral pulses were present, and blood pressure was 100/60 mmHg. She had mild pallor, otherwise general physical examination was normal. There was dry gangrene of left-hand fingers (except thumb) distal to metacarpophalangeal joints (Figure 1) with absent sensations. There were similar but milder findings on the second and fourth toes of the right foot. Systemic examination was normal.

Small vessel vasculitis was considered as there was thrombocytosis (platelet count was 640,000/mm^3^). But we proceeded first to rule out infections, sepsis, endocarditis, and coagulation abnormalities. Transthoracic echocardiography did not show any evidence of infective endocarditis. Cultures of blood and urine were sterile, and serum procalcitonin was normal. There were no active sediments or proteinuria in the urine. Disseminated intravascular coagulation screen, namely prothrombin time, fibrin degradation products, D-dimer, and peripheral smear, was normal. The patient did not have history of diabetes mellitus or hypertension. Serum lipid profile was normal and diabetes was ruled out by a normal glycated hemoglobin value (HbA1c). Funduscopy was normal and Schirmer’s test was negative. There was no fever recorded during hospital stay.

Because of the digital ischemia, an arterial thoracic outlet syndrome was also considered, but CT chest including angiography arteries of both upper limbs were normal. There were no imaging evidence of medium vessel vasculitis, such as polyarteritis nodosa, because CT angiography of abdominal vessels did not show microaneurysms in the celiac axis or renal vessels. Because of female gender, a connective tissue disease, such as systemic sclerosis and systemic lupus erythematosus, was strongly considered in the differential diagnosis, so an ANA test by indirect immunofluorescence was requested. As gangrene was progressing, she was given IV methylprednisolone 15 mg/kg bodyweight for five days. This was followed by oral prednisolone 1 mg per kg body weight. Monthly IV cyclophosphamide 15 mg/kg was also initiated, which she received for six cycles. Surgical team advised against any surgical intervention.

Meanwhile, results of ANA by indirect immunofluorescence returned positive +++, coarse speckled with cytoplasmic reticular staining pattern. Further immunological work-up was initiated. Antibodies to extractable nuclear antigens by line immunoassay was positive for anti-Ro and U1RNP. Anti-neutrophil cytoplasmic antibodies (ANCA) by indirect immunofluorescence was positive (++) with a peri-nuclear staining pattern (pANCA). However, myeloperoxidase and proteinase 3 antigen by commercial enzyme-linked immunoassay were negative. Antiphospholipid antibodies were negative, and serum complements were within normal range. Serum cryoglobulins were also negative. Serological markers for systemic sclerosis, namely anti-centromere antibodies and anti-topoisomerase 3 antibodies, could not be done due to non-availability of the facility in the hospital. Because of strongly positive ANA, anti-U1RNP, anti-Ro, and pANCA positivity, a diagnosis of evolving undifferentiated connective tissue disease (UCTD) was made. At more than one-year follow-up, the patient had had auto-amputation of the involved fingers. She continues to be on low-dose prednisolone, hydroxychloroquine, and azathioprine but still did not fulfill the criteria for any well-defined connective tissue disease.

## Discussion

The patient under description had digital gangrene that was otherwise unexplained by thrombus or atherosclerosis or infection or a thoracic outlet syndrome. It is clear that she has a connective tissue disease because the ANA, anti-U1RNP, anti-Ro antibodies, and pANCA were positive. Anti-Ro is present in Sjogren's syndrome and systemic lupus erythematosus, while pANCA is present in microscopic polyangiitis, crescentic glomerulonephritis, other autoimmune diseases like rheumatoid arthritis, and connective tissue diseases such as systemic lupus erythematosus. Antibodies to the Ui-70kDa small nuclear RNP (anti-U1RNP antibodies) are a defining feature of mixed connective tissue disease. Though anti-U1RNP was present, she had no qualifying features for a mixed connective tissue disease, such as Raynaud’s phenomenon, puffy fingers, arthritis, myositis, pulmonary hypertension, or interstitial lung disease. Some authors firmly believe that mixed connective tissue disease is not different from UCTD, and it is a precursor of systemic sclerosis [5].

Among the other connective tissue diseases systemic sclerosis is well known to present with digital ulcers and digital ischemia that can progress to gangrene [4]. But in our patient there were no features of systemic sclerosis, such as sclerodactyly, esophageal dilatation, or pulmonary fibrosis. There were also no abnormalities in the nail fold capillaroscopy.

Another consideration here is systemic lupus erythematosus. Digital gangrene developing during the course of lupus is well described. Jeffery et al. encountered seven patients with critical peripheral ischemia out of 487 lupus patients (1.4%) [4], while in an Indian series 20 out of 344 lupus patients (5.8%) seen over a 10-year-period had gangrene of digit or toe [6]. But our patient did not have any classical features of lupus, such as photosensitivity, malar rash, and discoid rash. She also did not have kidney involvement or serositis or arthritis. She also did not fit in a description of incomplete lupus [7]. For these reasons we made a diagnosis of UCTD. One could argue that ANA positivity is false positive, but then the extractable nuclear antigen was conclusively positive for other antibodies.

UCTD is a term used to describe patients presenting with clinical and serological manifestations of systemic autoimmune rheumatic diseases but not fulfilling the criteria for any defined connective tissue disease [8]. The most common manifestations of UCTD are arthralgia or arthritis, mild cytopenias, serositis, Raynaud’s phenomenon, and malar rash or photosensitivity [8]. These features are not present always in a single point of time. In most series, 30-35% of patients with UCTD will evolve to a well-defined connective tissue disease, most commonly systemic lupus erythematosus over a period of one to five years. Lupus is a disease that is typically characterized by the presence of multiple antibodies. Our patient also had multiple autoantibodies namely anti-U1RNP, anti-Ro, and pANCA but there were no other clinical features of lupus. 

A search of the literature in PubMed for “UCTD and gangrene” OR “UCTD and digital ischemia” OR “undifferentiated connective tissue disease and digital ischemia” retrieved two papers [9,10]. One paper described a patient similar to ours. This was a patient with gangrene of fingers, ANA positive +++ homogenous staining pattern, anti-Ro positive, with no other features of autoimmune disease. Like our patient she was managed with pulse methylprednisolone and cyclophosphamide [9]. The second paper was a description of 21 patients with connective tissue disease with moderate to high titers of anti-phospholipid antibodies [10]. In this series, one patient was classified as UCTD.

Digital gangrene in connective tissue could arise due to small vessel vasculitis, vasospasm of Raynaud’s phenomenon, microthrombi due to antiphospholipid syndrome, and/or accompanying accelerated atherosclerosis [3]. Most cases are managed with a combination of immunosuppressants, anticoagulants, and aspirin. The reason for presenting this case is because of the critical nature of the illness causing distress to the patient, but prompt initiation of immunosuppressive treatment led to arrest of progression of the gangrene. 

## Conclusions

The medical causes of digital gangrene range from infections, vasculitis, vascular occlusion by a thrombus, or a connective tissue disease. Good clinical history and examination are helpful to rule out infections, vasculitis, Raynaud's phenomenon, and many connective tissue diseases, such as lupus and systemic sclerosis. Imaging is needed to rule out thrombus in major arterial systems. When a connective tissue disease is suspected and other usual causes are reasonably ruled out, early treatment with glucocorticoids and immunosuppressant agents can arrest the progression of gangrene as exemplified by this case. 
